# Flexible Waterborne Polyurethane-Bacterial Cellulose Films for Real-Time Physiological Monitoring

**DOI:** 10.3390/polym17060787

**Published:** 2025-03-16

**Authors:** Jiujiang Ji, Changyong (Chase) Cao, Ruixiang Qu, Ningjing Zhou, Enjian He, Mingrui Wu, Huacui Xiang, Zhijun Ma, Guojun Liu, Yen Wei

**Affiliations:** 1 The Key Laboratory of Bioorganic Phosphorus Chemistry & Chemical Biology, Department of Chemistry, Tsinghua University, Beijing 100084, China; 2 Department of Mechanical and Aerospace Engineering, Case Western Reserve University, Cleveland, OH 44106, USA; 3 Zhejiang Lab, Hangzhou 311121, China; 4 Department of Chemistry, Queen’s University, 90 Bader Lane, Kingston, ON K7L 3N6, Canada

**Keywords:** bacterial cellulose (BC), waterborne polyurethane (WPU), flexible electrodes, wearable devices, electrocardiograph (ECG)

## Abstract

The incorporation of waterborne polyurethane (WPU) into bacterial cellulose (BC) fibers significantly improved the tensile strength of the resulting WPU/BC composite film, achieving an enhancement of 19.4 times. The formation of hydrogen bonds between WPU and BC effectively eliminates cavities within the BC matrix, achieving significant plasticization and toughening. Compared with the pure BC film (WPU/BC-0), the elastic modulus of the WPU/BC-5 composite film is reduced by 97.5%, and surface hardness is decreased by 96.9%. When integrated with a flexible EGaIn electrode, the wearable composite film demonstrated exceptional potential in flexible electronics, reliably enabling point-of-care detection of human electrocardiograph (ECG) signals. This WPU-regulated BC approach provides a promising alternative for fabricating flexible and durable substrates suitable for wearable device applications.

## 1. Introduction

Flexible films are widely used as substrate materials in wearable devices, implantable bioelectrodes, battery diaphragms, and related applications [1]. Over the past decades, soft, thin-film structures have emerged as a dominant platform for bioelectronics [2,3,4,5], driving the advancements in wearable technologies. The integration of bioelectronic systems on flexible substrates has spurred significant research and innovation, particularly in wearable devices [6,7,8]. However, most flexible films are currently derived from non-renewable, petroleum-based polymers, presenting environmental and sustainability challenges [9].

Cellulose, a natural polymer produced by plants or bacteria, stands out as an abundant, biodegradable, and renewable material [10]. Through physical and chemical processing, cellulose can be transformed into its derivatives, such as cellulose nanocrystals (CNC), cellulose nanofibers (CNF), and bacterial cellulose (BC) [11]. Among these, BC synthesized by bacteria has gained substantial attention due to its high crystallinity, low density, excellent water retention, polyhydroxylated interface, and renewability [11]. Despite these advantages, the strength and toughness of BC films are significantly lower than those of most engineering plastics, which limits their application [12]. Addressing the challenge of enhancing the mechanical properties of BC-based films remains a critical area of research [13,14].

BC’s unique three-dimensional (3D) porous network structure and polyhydroxylated properties make it an ideal candidate for designing functional composites [15], serving as flexible substrates or structural reinforcement materials [16]. However, BC films in their dry state exhibit limited flexibility and low elongation at break. Conventional strategies for modifying BC films, such as incorporating plasticizers like glycerol and glutaraldehyde [17,18], have improved their flexibility and stretchability to a certain degree. Nevertheless, these improvements remain inadequate for practical applications [19]. In order to use bacterial cellulose as a flexible material substrate in the field of wearable devices, its flexibility needs to be further enhanced. To utilize bacterial cellulose as a flexible material substrate in wearable devices, its flexibility must be further enhanced [20,21].

Waterborne polyurethane (WPU), an environmentally friendly flexible polymer, offers excellent post-modification capabilities due to the presence of hydrophilic groups (e.g., carboxyl, amino, silicon hydroxyl) introduced during its synthesis [22]. While WPU possesses many desirable properties, its mechanical strength is insufficient and requires enhancement [11]. Therefore, BC’s 3D framework can effectively reinforce WPU’s mechanical strength, while WPU can, in turn, improve the flexibility and toughness of the composite film [23,24], creating a synergistic effect. Thus, the combination of WPU and BC in the preparation of flexible substrate materials will expand its application range significantly.

In this study, a flexible and foldable WPU/BC film was developed by infiltrating the BC network with a WPU emulsion, forming a composite stabilized by hydrogen bonds. The BC’s porous network and polyhydroxyl properties synergized with WPU to produce a highly flexible composite material. The prepared WPU/BC substrate exhibited not only remarkable stretchability but also outstanding plasticization and toughening properties. To further enhance its functionality, an Ag layer was deposited onto the film using magnetron sputtering, followed by printing EGaIn onto the film to create an LM-Ag-WPU/BC electrode. The resulting flexible electrode demonstrated the capability to reliably detect electrocardiograph (ECG) signals. The WPU/BC composite film exhibited excellent flexibility, folding resistance, and suitability for wearable devices, offering promising potential in electronic and biomedical applications.

## 2. Materials and Methods

### 2.1. Materials

The aqueous bacterial cellulose (BC) suspension (6 wt%; source: *Komagataeibacter*; fiber size: 50–100 nm in diameter, >20 mm in length) was supplied by Songhu Shenjian Technology Co., Ltd. (Shenzhen, China). Waterborne polyurethane (WPU) was obtained from Macklin (40 wt%, Shanghai, China). Nylon filter membranes (1 μm and 5 μm) were acquired from Shanghai Xihe Technology Co., Ltd., Shanghai, China. All reagents were used as received without further purification.

### 2.2. Preparation of the WPU/BC Film and Flexible Electrode

To prepare the WPU/BC film, varying proportions of BC and WPU were added to 50 mL of aqueous solution and stirred at 60 °C for 6 h. The resulting mixture was initially filtered through a 5 μm nylon filter membrane, and an appropriate volume of the solution was then passed through a filtration device equipped with a 5 μm nylon filter membrane to yield the WPU/BC film. For the composite WPU/BC-5 film, 10 g of aqueous BC suspension, 10 g of WPU, and 10 g of deionized water were mixed and stirred for 6 h. After the mixture was filtered through a 5 μm nylon filter membrane, it was subsequently filtered through a 1 μm nylon filter membrane to obtain WPU/BC-5 films of varying thickness. The recipe for the WPU/BC film is provided in Table 1.

### 2.3. Fabrication of the LM-WPU/BC Electrode

The slightly adhesive tape, after being laser-engraved with pre-designed patterns, was closely adhered to the WPU/BC film as a masking layer. Subsequently, an Ag layer was deposited onto the film via magnetron sputtering to obtain Ag-WPU/BC, using a sputtering time of 10 min and a current of 20 mA. The LM-Ag-WPU/BC was then prepared by printing 50 μL of EGaIn onto the Ag-pretreated WPU/BC film in a glove box. Finally, the tape mask was removed, and the LM-Ag-WPU/BC was cleaned with ethanol to remove excess LM.

### 2.4. ECG Signal Acquisition Module

The picture of the Single-Lead Heart Rate Monitor Front End (AD8232, Analog Devices, Wilmington, MA, USA) is shown in Figure S5. The AD8232 is an integrated signal conditioning module specifically designed for ECG monitoring. This device extracts, amplifies, and filters weak ECG signals, even in the presence of noise caused by motion or remote electrode placement. Its design allows an ultra-low power analog-to-digital converter (ADC) or an embedded microcontroller to easily capture the output signal. The AD8232 incorporates a double-pole high-pass filter to eliminate motion artifacts and electrode half-cell potentials. This filter is closely integrated with the structure of the instrumentation amplifier, enabling single-stage high-gain and high-pass filtering.

### 2.5. Characterization

#### 2.5.1. Fourier-Transform Infrared (FTIR) Spectroscopy

The chemical structures of the WPU/BC film were measured by FTIR spectroscopy (VERTEX 80/80v, Bruker, Germany) in the range of 4000–400 cm^−1^. Each thin film was scanned 32 times at a resolution of 4 cm^−1^ in transmission mode.

#### 2.5.2. X-Ray Photoelectron Spectroscopy (XPS)

The chemical composition and surface properties of the WPU/BC film were analyzed using XPS (ThermoFisher Scientific, Waltham, MA, USA) with a Thermo ESCALAB 250Xi spectrometer using an Al Kα X-ray source (1486.6 eV) [25].

#### 2.5.3. Morphology Characterization

The morphology of the samples (WPU/BC-0 to WPU/BC-5) was investigated by SEM (SU-8010, Hitachi Limited, Tokyo, Japan).

#### 2.5.4. Water Contact Angle (WCA) Analysis

The hydrophobicity of the fabricated WPU/BC films was determined using a contact angle measurement instrument (OCA 15 machine, Data-physics, Filderstadt, Germany). A 5 μL syringe drop with deionized water was used for testing, and the results were reported as averages ± standard deviations [26].

#### 2.5.5. Thermal Properties

The maximum decomposition temperature of WPU/BC films was determined using a thermogravimetric analyzer (TGA-6, PerkinElmer, Waltham, MA, USA). In the thermal analysis, each film sample (approximate weight, 5 mg) was heated from 30 °C to 800 °C at a rate of 10 °C·min^−1^ under a nitrogen flow at a rate of 20 mL·min^−1^ [18].

#### 2.5.6. Recording of Human ECG Signal

The ECG signal collection using the LM-Ag-WPU/BC-5 as the electrode was conducted with an Arduino-based ECG testing module and commercial electrode patches. Prior to the test, the designated areas, the right shoulder below the clavicle, the left shoulder below the clavicle, and the left lower waist, were cleaned with alcohol-soaked cotton. When using LM-Ag-WPU/BC-5 as the acquisition end, the conductive hydrogel at the front of the commercial electrode patch was replaced with LM-Ag-WPU/BC-5 to capture the signals [27].

## 3. Results and Discussion

**Figure 1:**
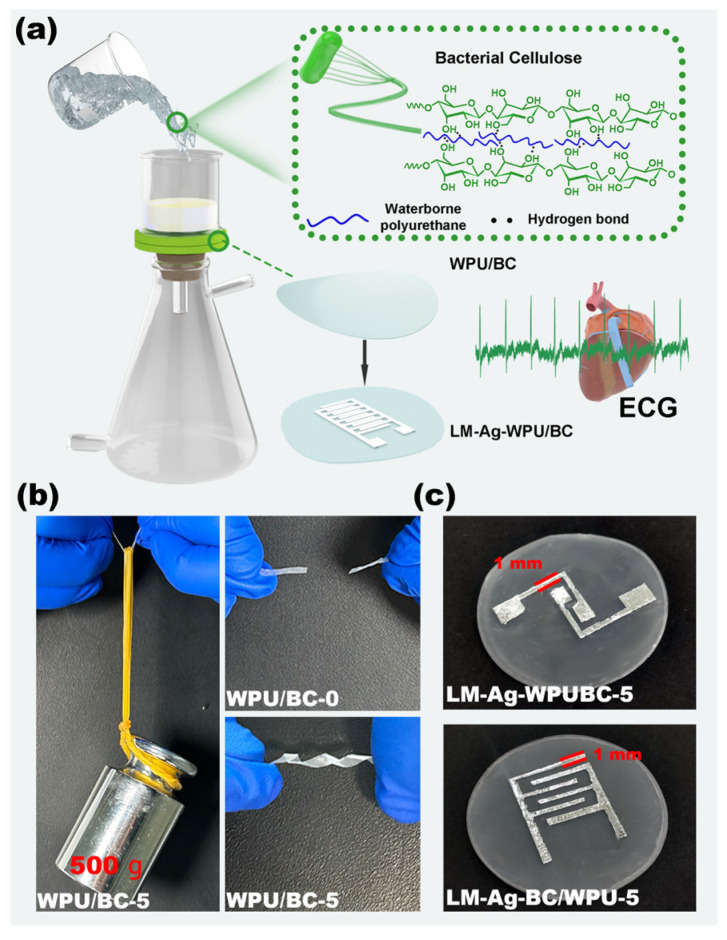
(**a**) Schematic diagram illustrating the preparation process of WPU/BC substrates and LM-WPU/BC flexible electrodes. (**b**) Photographs demonstrating the toughness and twistability of WPU/BC-0 and WPU/BC-5 films (dimensions: length 35 mm, width 2 mm, thickness 50 μm). (**c**) Images showing the LM-Ag-WPU/BC-5 electrodes.

The preparation process for the WPU/BC composite film is illustrated in Figure 1a. First, the BC suspension was thoroughly mixed. The solvent was then removed via vacuum filtration, and the mixture was dried at room temperature, resulting in a soft film with a dense structure. Photographs of the WPU/BC films (WPU/BC-0 to WPU/BC-5) fabricated using this method are shown in Figure S1. The modified composite film exhibited sufficient flexibility to serve as a substrate for flexible electrodes, enabling real-time ECG monitoring. As depicted in Figure 1b, the synthesized WPU/BC-5 film (length: 35 mm; width: 2 mm; thickness: 50 μm) could easily support a 500 g weight, whereas the WPU/BC-0 film of the same dimensions failed under a 100 g load. Furthermore, the WPU/BC-5 film demonstrated remarkable flexibility, toughness, and resilience, tolerating complex distortions and exhibiting elastomer-like properties. After the magnetron sputtering an Ag layer onto the WPU/BC-5 film, EGaIn was firmly deposited to create LM-Ag-WPU/BC-5 electrodes, as shown in Figure 1c.

**Figure 2:**
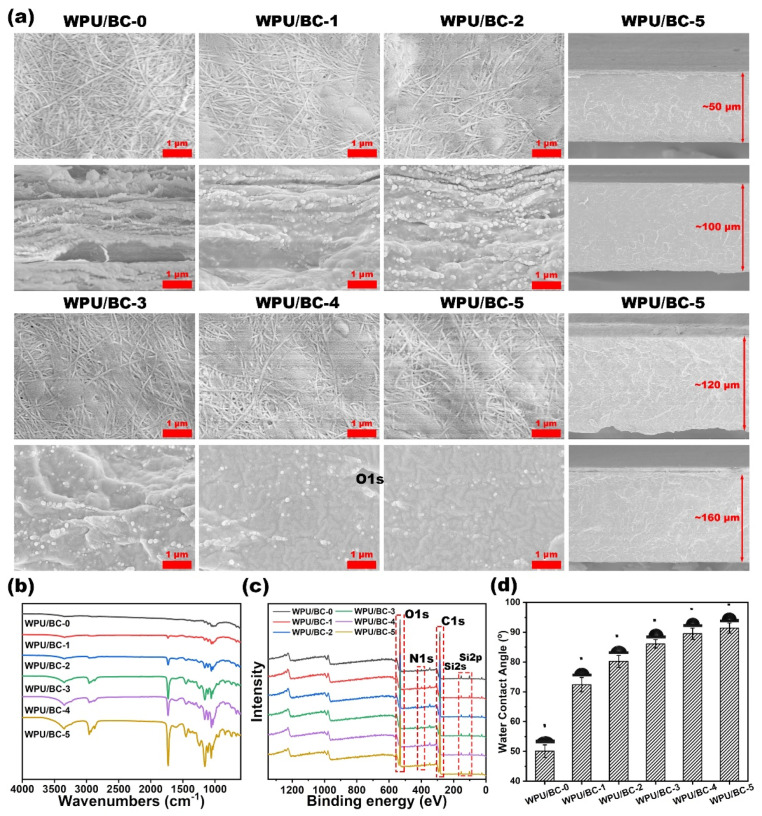
(**a**) SEM images of WPU/BC-0 to WPU/BC-5 films showing surface (**top**) and cross-section (**bottom**) morphologies, along with cross-sectional images of WPU/BC-5 films with varying thicknesses. (**b**) FTIR spectra of WPU/BC-0 to WPU/BC-5 films. (**c**) XPS analysis of the surfaces of WPU/BC-0 to WPU/BC-5 films. (**d**) Water contact angle measurements for the surfaces of WPU/BC-0 to WPU/BC-5 films.

To validate the effectiveness of the fabrication strategy, the surface and cross-sectional topography of WPU/BC-0 to WPU/BC-5 films were analyzed using scanning electron microscopy (SEM) [17]. As shown in Figure 2a, the SEM images of the WPU/BC-0 film displayed a random distribution of nanofibers. In contrast, localized cross-linking phenomena were evident on the surfaces of WPU/BC-1 to WPU/BC-5, indicating changes in surface morphology across the composite films. The surface structure became increasingly dense with higher WPU content. Cross-sectional SEM images of the WPU/BC composite films further revealed that the film cross-sections also became denser as the WPU addition increased, reflecting the improved compactness of the composite structure. The thickness of the WPU/BC composite films could be controlled during the vacuum filtration process, producing films with thicknesses of 80 μm, 100 μm, 120 μm, and 160 μm. This control capability demonstrates the versatility of the preparation method. Notably, reducing the substrate thickness lowered bending rigidity, thereby enhancing conformal contact with surfaces.

The FTIR spectra of WPU/BC-0 to WPU/BC-5 films, shown in Figure 2b, confirmed the integration of BC and WPU. The characteristic absorption peaks at 3345 cm^−1^ and 1065 cm^−1^ corresponded to the stretching vibration of the O–H and C–O groups, respectively [22]. Peaks at 2854 cm^−1^ and 2930 cm^−1^ were attributed to the stretching vibrations of C–H. Additionally, the peaks at 1530 cm^−1^ and 1729 cm^−1^ represented the stretching vibrations of C=O and the deformation vibration of C–N, respectively, further confirming the presence of WPU in the composite.

As shown in Figure 2c and Figure S2, the C1s spectrum revealed three peaks at 284.8 eV, 286.5 eV, and 288.2 eV, corresponding to C–C, C–O, and C=O bonds, respectively [11]. Notably, the relative strength of the C–C peak increased to 49.44%, while the C=O peak strength decreased to 14.92%. This shift indicates the formation of numerous hydrogen bonds between BC and WPU, confirming that WPU effectively modified the BC film. Additionally, it was demonstrated that the WPU/BC substrates prepared by this method exhibited good uniformity.

Pure BC films, while inherently hydrophilic due to their rough fiber surface and abundance of hydroxyl functional groups, present challenges in wearing comfort. The incorporation of WPU introduced surface hydrophobicity, significantly enhancing the wearability of the substrate. As shown in Figure 2d, the unmodified WPU/BC-0 film exhibited a water contact angle (WCA) of 50.1° ± 2.1°. In contrast, the WPU-modified films demonstrated a substantial increase in WCA with higher WPU content, peaking at 91.4° ± 1.7° for the WPU/BC-5 film. This improved hydrophobicity enhanced the comfort and wearability of the WPU/BC substrates, supporting their potential application in wearable devices [11].

The mechanical mismatch between rigid electrodes and soft tissues often results in electrode damage and chronic inflammatory responses due to the direct contact required with biological tissues. Soft substrates for electrodes, however, can achieve seamless contact with the skin, effectively mitigating motion-induced artifacts [28]. Compared to conventional rigid, bulky, and planar electronics, soft electrodes enable conformal and compliant attachment to soft, dynamically deformable, and irregularly shaped organs, such as skin, brain, and joints. Due to the excellent properties of the flexible substrates, they do not cause damage to the skin of the organism in practical applications.

**Figure 3:**
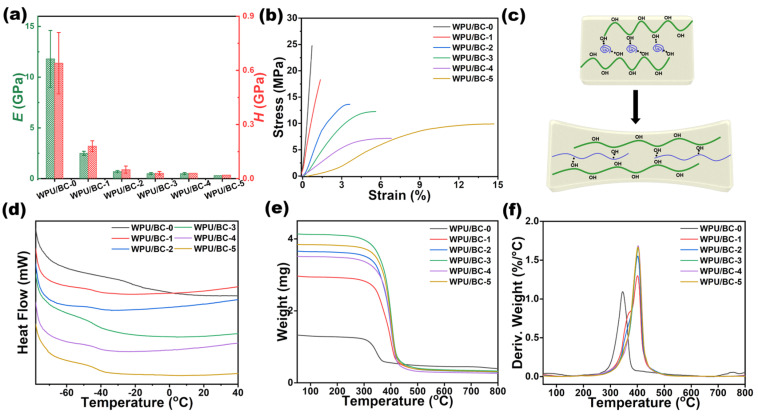
(**a**) Equivalent elastic modulus (E) and surface hardness (H) of WPU/BC-0 to WPU/BC-5 films. (**b**) Stress–strain curves of BC/WUP films before and after stretching. (**c**) Schematic illustration of the stretchable network of BC/WUP composite. (**d**–**f**) DSC, TGA, and DTG curves of the pristine BC and WPU/BC composite films.

Figure 3a presents the surface hardness of WPU/BC-0 to WPU/BC-5 films obtained from nanoindentation experiments. The equivalent elastic modulus of the films was calculated, revealing a significant reduction with increasing WPU content. The elastic modulus of WPU/BC-0 was 11.8 ± 2.8 GPa, which decreased by 97.5% to 0.3 ± 0 GPa in WPU/BC-5. Similarly, the surface hardness of WPU/BC-0 was 0.64 ± 0.17 GPa and decreased sharply to 0.02 ± 0 GPa (96.9% reduction) in WPU/BC-5. These results demonstrate that the addition of WPU significantly enhances the softness of the composite film.

Figure 3b shows the stress–strain curves of WPU/BC-0 to WPU/BC-5 films. The WPU/BC-5 film exhibited a tensile strength of 9.91 MPa and an elongation at break of 14.71%, representing a 19.4-fold improvement in tensile strength compared to WPU/BC-0 (0.72%). However, as the WPU content increased, the tensile strength of the composite film decreased. This decline was attributed to the reduced relative content of BC, weakening the BC skeleton and adversely affecting its mechanical properties.

The toughening mechanism of the WPU/BC composite film is presented in Figure 3c. Hydrogen bonds formed between WPU and BC effectively eliminated void defects within the BC matrix, achieving plasticizing toughening. Introducing WPU into the BC matrix created hydrogen bonds or other interactions within the BC molecular chain, enhancing the flexibility of the composite film. At the molecular level, the homogeneous dispersion of WPU maintained the internal network structure of BC and strengthened the connections between molecular segments through hydrogen bonding, thereby improving the tensile properties of the composite film.

As depicted in Figure 3d, the differential scanning calorimeter (DSC) curve revealed that the glass transition temperature (T_g_) of WPU/BC-0 was approximately −22 °C, while the T_g_ of WPU/BC-1 to WPU/BC-5 decreased to about −43 °C. The relatively low T_g_ ensured the flexibility of this composite substrate at both room and low temperatures, making it superior to most existing biomass membrane substrates and suitable for use in extreme temperature environments.

The thermal stability of BC and WPU/BC composite films was further analyzed using thermogravimetric analysis (TGA) and differential thermal analysis (DTA), as shown in Figure 3e,f and Figure S3. Pure BC exhibited a single distinct weight loss peak on the DTG curve at around 350 °C, corresponding to the thermal decomposition of BC. In contrast, the WPU/BC-1 composite film displayed two thermal degradation peaks. The first peak at 380 °C was primarily attributed to the thermal decomposition of BC, while the second peak at approximately 400 °C was due to the decomposition of WPU. As the WPU content increased and the BC content decreased, the thermal stability of the WPU/BC composite films improved, meeting the application requirements for flexible substrates across various application scenarios.

Preliminary data suggest that electrophysiological methods can extract valuable information about potential myocardial disease and translate these signals into models capable of controlling external devices [28]. As more patients benefit from this approach, interest in flexible wearable devices has grown significantly. However, rigid substrates often fail to conform to the human body, causing significant interference with collected signals. Soft and flexible substrate materials effectively address these limitations [11].

**Figure 4:**
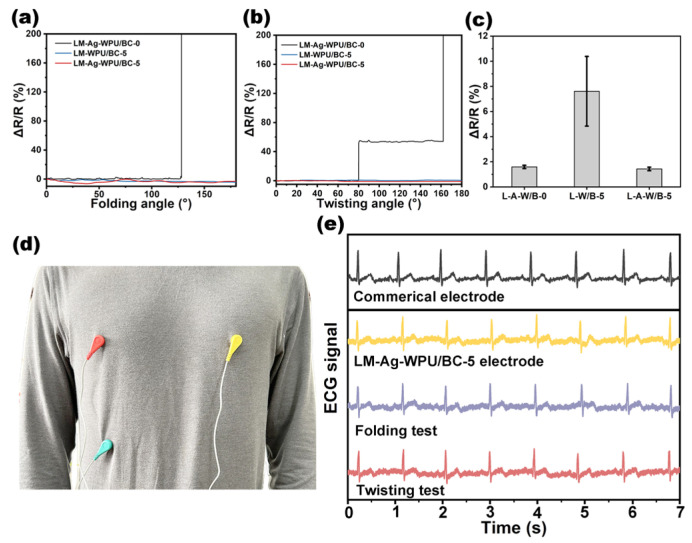
(**a**–**c**) Resistance changes in LM-Ag-WPU/BC-0, LM-WPU/BC-5, and LM-Ag-WPU/BC-5 after folding, twisting, and immersion tests. (**d**) Digital images of three LM-Ag-WPU/BC-5 flexible electrodes integrated into a setup for ECG signal collection. (**e**) ECG signals recorded by commercial electrodes compared to those collected by LM-WPU/BC-5 electrodes, as well as ECG measurements using LM-WPU/BC-5 electrodes after repeated bending and twisting tests.

The electrical stability of the flexible electrodes during deformation was evaluated. Resistance changes in LM-Ag-WPU/BC-0, LM-WPU/BC-5, and LM-Ag-WPU/BC-5 during folding and twisting are shown in Figure 4a,b. For the rigid BC substrate (WPU/BC-0), poor flexibility resulted in substantial resistance changes during deformation. In contrast, the WPU/BC-5 substrate demonstrated excellent flexibility, maintaining stable resistance under similar conditions.

To assess immersion stability, the electrodes were soaked in a sterile 0.9% sodium chloride solution for 48 h. The resistance changes are depicted in Figure 4c. The LM-Ag-WPU/BC-5 electrodes, with an Ag layer added via magnetron sputtering, exhibited minimal resistance change during immersion, highlighting the strong bonding between EGaIn and the substrate. Conversely, electrodes without the Ag layer displayed significant resistance changes, emphasizing the advantage of the proposed fabrication method. This also demonstrated that the prepared LM-Ag-WPU/BC-5 can maintain close contact with the skin without causing penetrative damage [27]. Additionally, the adhesion stability of the electrodes was evaluated. Repeated tear tests demonstrated a significant increase in the resistance of LM-WPU/BC-5 after 10 cycles, while LM-Ag-WPU/BC-5 exhibited minimal change under cyclic tearing (Figure S4).

As shown in Figure 4d, the flexible LM-Ag-WPU/BC-5 electrodes were integrated into a T-shirt, enabling stable ECG signal collection. Compared to commercial electrodes, these flexible LM-Ag-WPU/BC-5 electrodes could reliably collect ECG signals, even after 200 cycles of folding and bending, with no significant noise or distortion observed in the ECG waveforms (Figure 4e). In contrast, slight folds or distortions at the test site caused remarkable fluctuations in the LM-Ag-WPU/BC-0 electrode (Figure S6), underscoring the benefits of flexible substrates for wearable applications.

## 4. Conclusions

This study introduced a novel strategy for developing a flexible substrate by infiltrating WPU into the 3D network of BC. The formation of hydrogen bonds between WPU and BC effectively eliminated internal voids within the BC matrix, achieving significant plasticization and toughening. As a result, the elastic modulus of the WPU/BC-5 composite film decreased by 97.5%, and surface hardness reduced by 96.9% compared to pure BC film (WPU/BC-0). Notably, the tensile strength of the WPU/BC-5 film was enhanced by 19.4 times relative to the WPU/BC-0 film. The resultant LM-Ag-WPU/BC electrode demonstrated excellent performance in point-of-care detection of human ECG signals. This work provided a promising approach for designing flexible substrates, paving the way for advancements in wearable electronic devices.

## Figures and Tables

**Table 1:** The recipe of WPU/BC film.

WPU (6 wt%, g)	BC (6 wt%, g)	H_2_O (g)	Mixture (g)	Samples
0	10	10	10.0	WPU/BC-0
2	10	10	9.2	WPU/BC-1
4	10	10	8.6	WPU/BC-2
6	10	10	8.1	WPU/BC-3
8	10	10	7.8	WPU/BC-4
10	10	10	7.5	WPU/BC-5

## Data Availability

The data supporting this article have been included as part of the ESI.

## Supplementary Materials

The following supporting information can be downloaded at: https://www.mdpi.com/article/10.3390/polym17060787/s1, Figure S1: Photographs of WPU/BC-0 to WPU/BC-5 films fabricated using the vacuum filtration method. Figure S2: Spectra of densified C1s of WPU/BC-0 (a) and WPU/BC-5 films (b). Figure S3: TG-DTA curves of the pristine BC and WPU/BC composite films. Figure S4: Resistance changes of LM-WPU/BC-5 and LM-Ag-WPU/BC-5 under repeat tearing. Rubbing and tearing test: Rubbing and tearing tests were conducted to study the durability of the LM-Ag-pSBS. (A piece of 3M transparent tape was carefully applied to the sample and pressed with a 100 g iron block for 30 s. Subsequently, the tape was peeled off at a rate of approximately 1 cm s^−1^. The resistance of the sample was recorded after each tear cycle). Figure S5: Photograph of the Single-Lead, Heart Rate Monitor Front End (AD8232, Analog Devices, USA) Figure S6: ECG signals collected by the LM-Ag-WPU/BC-0 electrodes and after repeated bending (a) and twisting (b) tests.
