# Supplementary material for: Flexible Waterborne Polyurethane-Bacterial Cellulose Films for Real-Time Physiological Monitoring
Source: Polymers (Basel). 2025 Mar 16;17(6):787. doi: 10.3390/polym17060787 (PMC11945170; doi:10.3390/polym17060787)
Supplement: Supplementary file 1 [file polymers-17-00787-s001.zip › polymers-3521011-supplementary.pdf]

Flexible Composite Films of Waterborne Polyurethane and Bacterial Cellulose for Real-Time Physiological Monitoring

Jiujiang Ji [1], Changyong (Chase) Cao [2], Ruixiang Qu [3, *], Ningjing Zhou [3], Enjian He [1], Mingrui Wu [1], Huacui Xiang [1], Zhijun Ma [3], Guojun Liu [4, *], Yen Wei [1, *]

[1.] *The Key Laboratory of Bioorganic Phosphorus Chemistry & Chemical Biology, Department of Chemistry, Tsinghua University, Beijing 100084, China*

[2.] *Department of Mechanical and Aerospace Engineering, Case Western Reserve University, Cleveland, OH, 44106, USA*

[3.] *Zhejiang Lab, Hangzhou 311121, China*

[4.] *Department of Chemistry, Queen's University, 90 Bader Lane, Kingston, Ontario K7L 3N6, Canada*

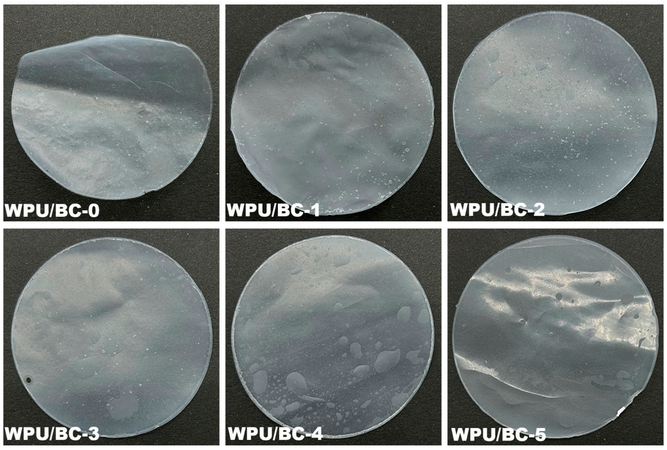

Fig. S1. Photographs of WPU/BC-0 to WPU/BC-5 films fabricated using the vacuum filtration method.

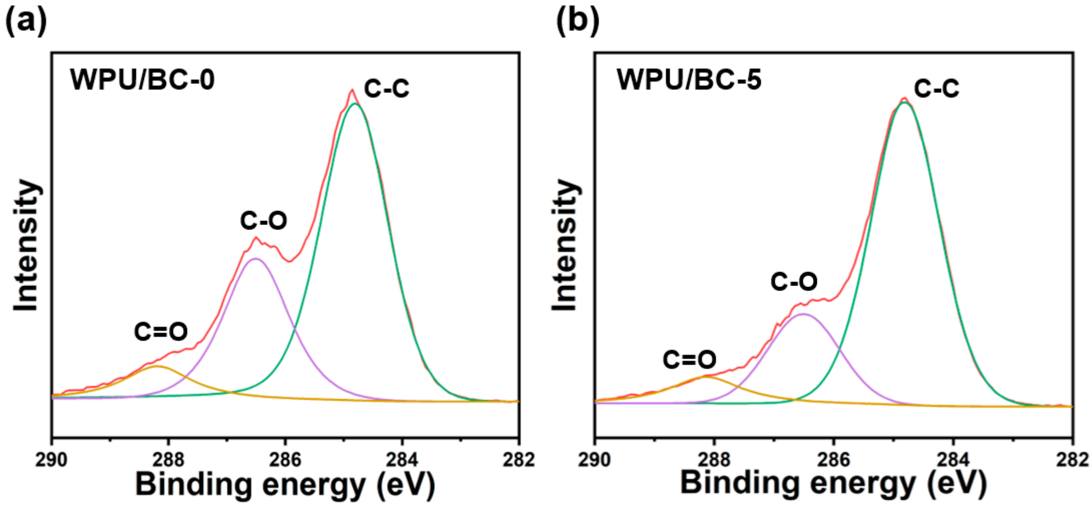

Fig. S2. Spectra of densified C1s of WPU/BC-0 (a) and WPU/BC-5 films (b).

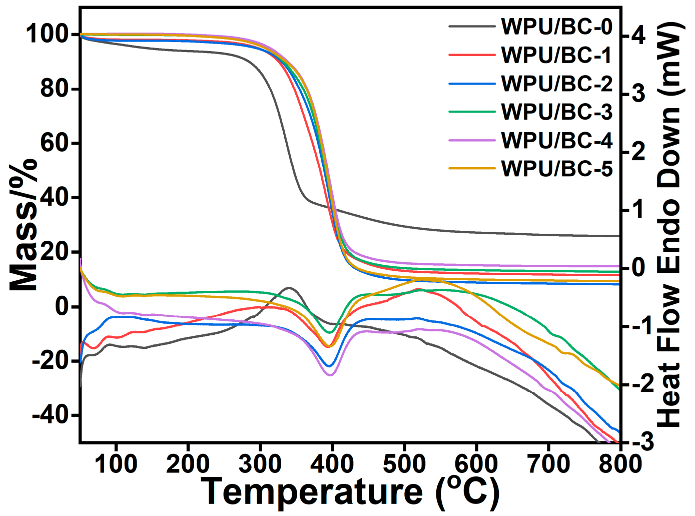

Fig. S3. TG-DTA curves of the pristine BC and WPU/BC composite films.

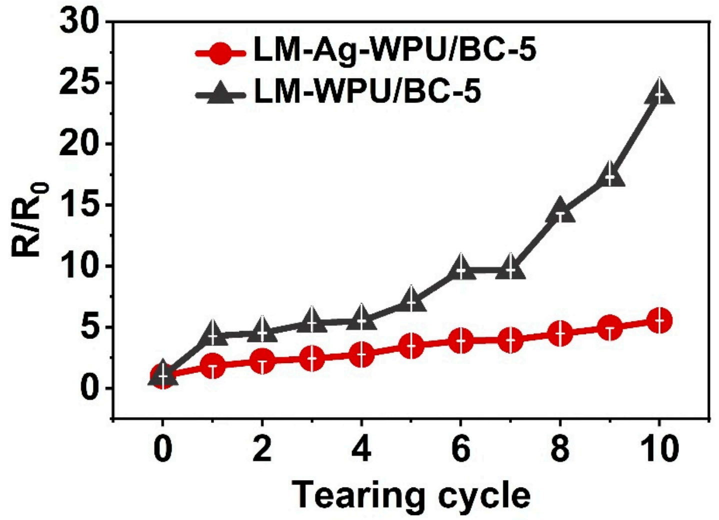

Fig. S4. Resistance changes of LM-WPU/BC-5 and LM-Ag-WPU/BC-5 under repeat tearing. Rubbing and tearing test: Rubbing and tearing tests were conducted to study the durability of the LM-Ag-pSBS. (A piece of 3M transparent tape was carefully applied to the sample and pressed with a 100 g iron block for 30 seconds. Subsequently, the tape was peeled off at a rate of approximately 1 cm s^{-1}. The resistance of the sample was recorded after each tear cycle.)

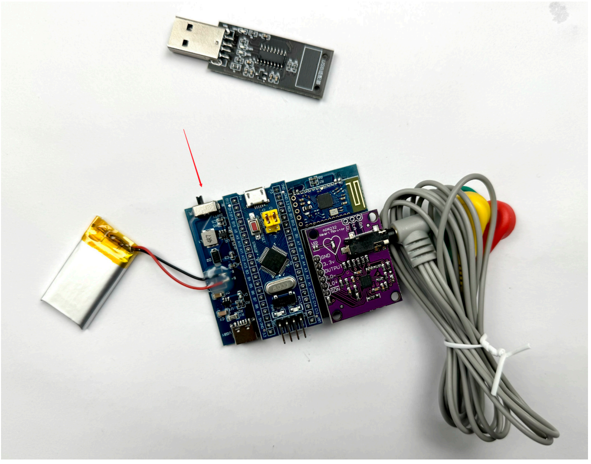

Fig. S5. Photograph of the Single-Lead, Heart Rate Monitor Front End (AD8232, Analog Devices, USA)

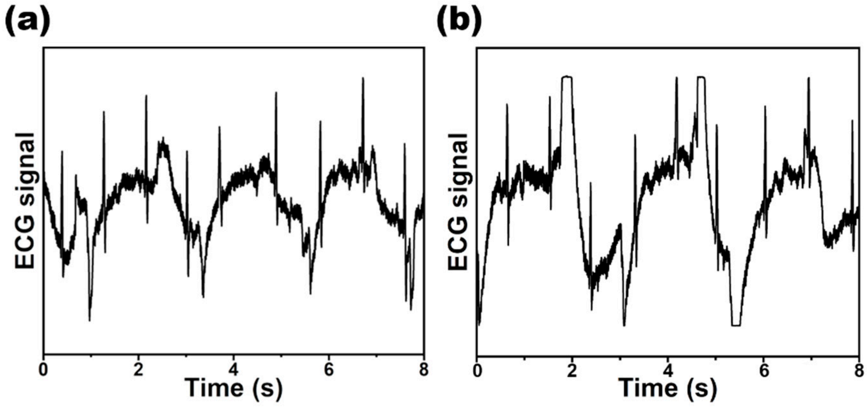

Fig. S6. ECG signals collected by the LM-Ag-WPU/BC-0 electrodes and after repeated bending (a) and twisting (b) tests.
